# Fabrication Routes, Microstructural Evolution, and Creep Performance of Oxide Dispersion Strengthened Austenitic Steels: A Review

**DOI:** 10.3390/ma19194159

**Published:** 2026-09-29

**Authors:** Yongbin Wang, Chenxin Yin, Zhangjian Zhou, Wenyue Zheng, Zhi Tong, Jinbao Wang

**Affiliations:** 1 School of Materials and Engineering, University of Science and Technology Beijing, Beijing 100083, China; 2 National Center for Materials Service Safety, University of Science and Technology Beijing, Beijing 100083, China; 3 Hebei Zhangxuangaoke Technology Co., Ltd., Zhangjiakou 075100, China

**Keywords:** oxide dispersion strengthened austenitic steel, mechanical alloying, process control agent, in situ internal oxidation, microstructure, mechanical properties

## Abstract

Oxide dispersion strengthened (ODS) austenitic steels exhibit superior high-temperature stability and mechanical properties, which are attributed to the strong pinning effect of nano-oxide particles on dislocations and grain boundaries. The microstructure and properties of ODS steels are intrinsically governed by their fabrication techniques. While powder metallurgy (PM) serves as the predominant synthesis route, the fabrication of austenitic ODS steels faces distinct technical challenges compared to their ferritic counterparts, primarily due to issues such as powder sticking to the milling media. Conversely, although traditional melting processes often lead to oxide agglomeration, coarsening, or flotation, which makes it difficult to achieve a uniform dispersion, they remain of significant interest because of their scalability and cost-effectiveness. Furthermore, emerging technologies such as additive manufacturing (AM) have also been employed for the preparation of ODS steels. This review provides a systematic and comprehensive overview of the recent progress in the fabrication technologies of ODS austenitic steels. The scope encompasses a critical analysis of the merits and limitations of techniques including PM, in situ internal oxidation, melting, and AM, alongside an examination of the potential impacts of adding process control agents (PCA) to mitigate powder sticking during mechanical alloying. Additionally, the microstructural characteristics resulting from different processing routes are discussed in detail, followed by a consolidated evaluation of their mechanical properties.

## 1. Introduction

Oxide dispersion strengthened (ODS) steels are considered for nuclear and other high-temperature applications because nanoscale oxides can improve their strength and microstructural stability under demanding service conditions [1,2,3]. Among them, ODS austenitic steels are attractive candidate structural materials because they retain the corrosion resistance and engineering maturity of austenitic stainless steels while benefiting from the strong pinning effect of finely dispersed nano-oxides [4]. These particles impede dislocation motion, grain-boundary migration, and substructural recovery during high-temperature exposure, thereby enhancing strength, creep resistance, and microstructural stability [5,6].

Conventional melting routes are generally incapable of achieving a uniform nanoscale dispersion of oxide particles within the metallic matrix [7]. Externally introduced oxides tend to agglomerate, coarsen, or float in the melt, leading to an inhomogeneous distribution of dispersoids and, consequently, instability in both microstructure and properties [8,9]. Therefore, the powder metallurgy route remains the most mature and widely adopted approach for fabricating ODS austenitic steels [10]. In this process, mechanical alloying (MA) promotes the refinement, dispersion, and even partial dissolution of oxide particles through repeated cold welding, fracturing, and high-energy mechanical impacts [11]. The mechanically alloyed powders are subsequently consolidated into bulk ODS materials by techniques such as hot pressing (HP) [12], hot extrusion (HE) [13], hot isostatic pressing (HIP) [14], or spark plasma sintering (SPS) [15]. In recent years, emerging processing strategies, including in situ internal oxidation [16] and additive manufacturing (AM) [17], have also attracted increasing attention. These approaches are expected to mitigate the inherent limitations of conventional powder metallurgy, such as high energy consumption, long processing routes, limited scalability, and insufficient microstructural homogeneity [18].

From a microstructural perspective, ODS austenitic steels typically exhibit an ultrafine-grained structure, whose stability is maintained by finely dispersed nano-oxide particles through grain-boundary pinning [19]. The composition, crystal structure, size distribution, and number density of the dispersoids are strongly dependent on the alloying-element stoichiometry and processing route. For complex oxide systems such as Y-Zr-O [20], Y-Ti-O [21], Y-Al-O [22], and Y-Hf-O [23], the interfacial structure, coherency relationship, and thermal stability of the oxide/matrix interface play critical roles in determining strengthening efficiency, irradiation resistance, and high-temperature microstructural stability. Although previous studies have demonstrated that ODS austenitic steels exhibit pronounced advantages in room-temperature strength [19] enhanced elevated-temperature yield strength arising from oxide-, grain-boundary-, and dislocation-strengthening contributions [24], considerable uncertainty remains regarding their long-term creep behavior [6]. In particular, under prolonged high-temperature service conditions, oxide-particle coarsening [25], the formation of incoherent interfaces, and the heterogeneous distribution of dispersoids may degrade rupture strength and microstructural stability [6]. These issues represent key challenges that must be addressed for the engineering application of ODS austenitic steels.

This review focuses on the formation and evolution of nano-oxide particles in ODS austenitic steels, the processing control of dispersoid distribution, and the relationship between oxide-particle characteristics and high-temperature mechanical properties, with particular attention to creep behavior, aiming to provide guidance for alloy design and long-term performance optimization. For this focused narrative review, relevant publications were identified through searches of the Web of Science Core Collection, supplemented by backward and forward citation tracking of key studies and previous reviews. The literature search used combinations of terms related to ODS austenitic steels, mechanical alloying, process control agents, casting, additive manufacturing, microstructure, tensile properties, and creep. Peer-reviewed studies directly addressing the fabrication, microstructure, and mechanical performance of ODS austenitic steels were prioritized, and the selected literature was organized and comparatively assessed according to fabrication route, processing conditions, microstructural characteristics, and mechanical performance.

## 2. Fabrication Routes for ODS Austenitic Steels

### 2.1. Mechanical Alloying

The PM route represents the most mature and widely utilized approach for the preparation of ODS steels. Its core process involves employing MA to uniformly disperse nano-oxides within the metal powder matrix, followed by consolidation and densification treatments to yield dense bulk materials. MA is a characteristic non-equilibrium solid-state process driven by high-energy mechanical forces, in which materials undergo severe plastic deformation, fracturing, and cold welding, ultimately achieving homogeneous mixing at the atomic scale.

The performance of ODS austenitic steels prepared via the PM route is critically dependent on the quality and homogeneity of the precursor powders. The preparation of powders not only influences the dispersion state of oxide particles but also directly correlates with the microstructural densification and the realization of mechanical properties during the subsequent sintering process. Furthermore, the type of initial powder is equally critical. Employing pre-alloyed powders as a substitute for elemental powders can effectively reduce the specific surface area and minimize oxygen pickup, thereby enhancing the comprehensive properties of the final product [26]. Currently, austenitic ODS steels typically utilize gas-atomized pre-alloyed stainless steel powders, such as 304, 310, 316, and 316L, as raw materials. Varying contents of oxide powders like Y_2_O_3_ [11] and ZrO_2_ [27] as well as alloying powders such as Ti [28], Zr [29,30], and Al, are added to the matrix. The pre-alloyed or elemental powders are subjected to MA with Y_2_O_3_, followed by densification via methods such as HP, HE, HIP, or SPS.

#### 2.1.1. Effects of Ball Milling Process Parameters

High-energy ball milling is the principal technique used to achieve mechanical alloying. The selection between dry and wet milling depends on the powder system and the required balance among particle refinement, cold welding, and contamination control [31]. Key milling parameters, including the ball-to-powder ratio, milling time, milling speed, and milling environment, strongly affect the particle size, powder morphology, crystallite size, lattice strain, and phase constitution of mechanically alloyed powders [32].

**Figure 1:**
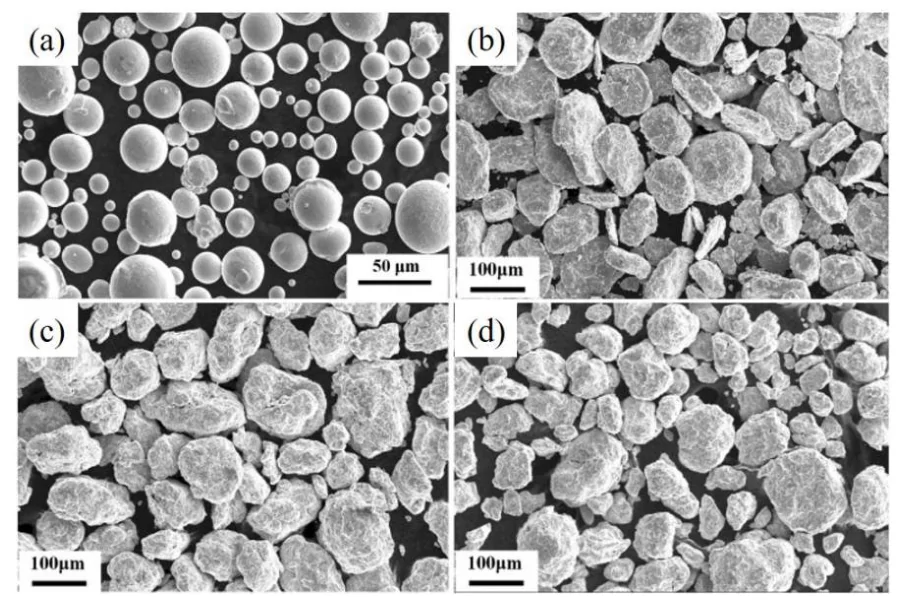
SEM images of powders after different milling times. (**a**) 0 h, (**b**) 5 h, (**c**) 30 h, (**d**) 50 h [11].

Research indicates [32] that appropriately increasing the BPR (e.g., from 6:1 to 12:1) and the milling speed to approximately 75% of the critical speed significantly enhances the collision energy and frequency. The critical speed is the theoretical rotational speed at which the centrifugal force keeps the milling balls continuously attached to the inner wall of the vial, preventing them from falling and effectively impacting the powder. Therefore, the operating speed is generally maintained below the critical speed to ensure effective impact during mechanical alloying. This, in turn, promotes particle refinement and grain nano-crystallization, while providing a higher driving force for solid-state phase transformations, thereby enabling the controllable regulation of powder characteristics [32]. Oka et al. [33] reported an appropriate milling-time window of 24–72 h for PNC316-based ODS austenitic stainless steel, within which chemical homogenization was achieved without detectable impurity-cluster formation. The exact milling time is dependent on the material composition, oxide content, ball-to-powder ratio, milling speed, and equipment; therefore, this numerical range cannot be directly applied to other materials, although the underlying optimization criterion provides a useful reference for other mechanically alloyed ODS systems. Figure 1 shows the evolution of powder morphology with milling time. As shown in Figure 1a, the initial powders exhibited a predominantly spherical morphology. After 5 h of milling, the powders were flattened into flake-like particles with an average diameter of approximately 100 μm owing to repeated collisions among the balls, powders, and vial, as shown in Figure 1b. With the milling time extended to 30 h, repeated cold welding led to the formation of larger and more rounded particles with a size of approximately 200 μm (Figure 1c). After 50 h of milling, work hardening and strain accumulation promoted powder fracturing, reducing the size of most particles to approximately 20 μm, as shown in Figure 1d. During the MA process, strain accumulation and work hardening lead to an increase in the brittleness of the powders, thereby facilitating further particle refinement. Gräning et al. [34] employed zirconia media, which not only effectively mitigated the issue of powder sticking but also achieved the controllable in situ introduction of Zr. The introduced Zr acted synergistically with Y_2_O_3_ and titanium oxides during the subsequent heat treatment to form Y-Zr-Ti-O complex nano-oxides, significantly enhancing the thermal stability of the dispersed strengthening phases.

To further optimize the formation mechanism of strengthening phases, Shi et al. [35] proposed a novel approach termed “reactive-inspired ball-milling” (RBM). Distinct from the conventional MA route, which is primarily dominated by physical fracturing, RBM utilizes mechanical energy to induce an in situ displacement reaction between Y and iron oxide, thereby directly generating Y_2_O_3_ nanoparticles within the matrix. Compared to traditional MA, this atomic-scale in situ synthesis mechanism yields nano-strengthening phases characterized by finer particle size, superior uniformity in distribution, and more stable interfacial bonding with the matrix, representing a significant developmental direction in high-energy ball milling technology.

#### 2.1.2. Phase Transformation Behavior During Ball Milling

During the MA process, the accumulation of high strain energy and the formation of a high density of lattice defects collectively constitute a potent driving force for solid-state phase transformations [36]. The stability of the austenite phase is not invariant during MA; the transformation of the austenite matrix (γ-Fe, fcc) into martensite (α′-Fe, bcc) is commonly observed during mechanical alloying [11]. Consequently, an in-depth understanding and effective regulation of this deformation-induced transformation behavior are of critical significance for controlling the microstructure of MA powders, optimizing the subsequent consolidation processes, and predicting the final material properties.

Numerous studies have indicated that during the mechanical alloying (MA) of Fe-Ni based powders, the fcc structure may transform into a bcc structure [37,38]. Similarly, in the investigation of the Fe-18Cr-8Ni system, Kotan [29] observed that the as-milled material was predominantly composed of α′-martensite, with the grain size refined to 20–25 nm. This clearly demonstrates that the immense strain accumulated during ball milling serves as the direct cause driving the γ→α′ phase transformation.

**Figure 2:**
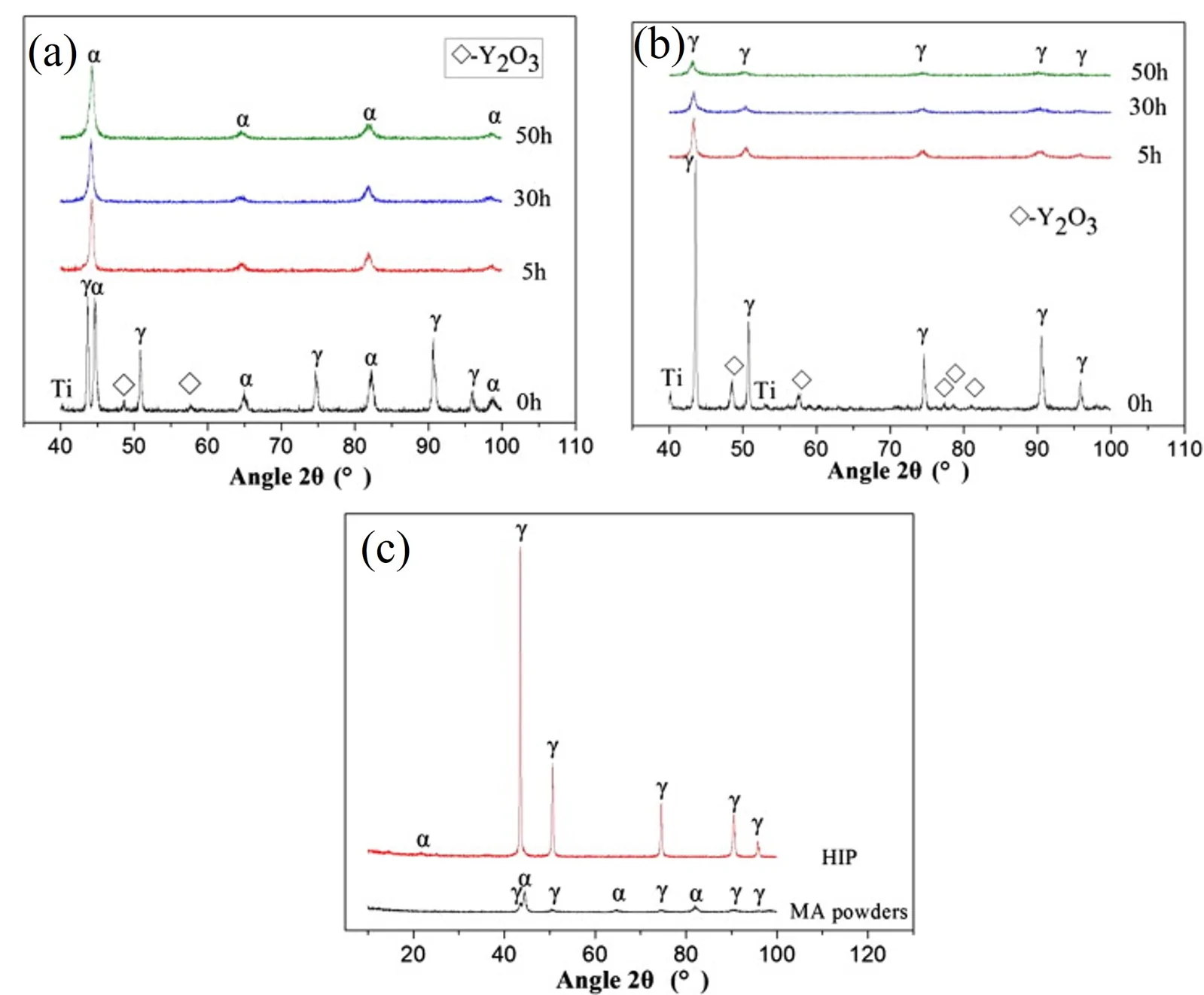
XRD pattern of the milled powders. (**a**) 304 (**b**) 310, (**c**) 316 [11].

This transformation is also critically dependent on the initial chemical composition, particularly the contents of key alloying elements such as Cr and Ni. Wang et al. [11] systematically investigated the phase transformation behavior of ODS-304, ODS-316, and ODS-310 during mechanical alloying. As shown in Figure 2, ODS-304 transformed almost completely into α′ martensite after milling (Figure 2a), ODS-310 retained a single γ phase because of its higher Ni content (Figure 2b), and ODS-316 exhibited a γ + α′ dual-phase structure (Figure 2c). These results indicate that increasing the Ni content stabilizes austenite and suppresses the strain-induced γ→α′ martensitic transformation, whereas ferrite-stabilizing elements such as Cr and Ti reduce austenite stability and thereby favor this transformation. This trend is further supported by the study of Enayati et al. [36], who observed that alloys with Ni contents of 8% and 15% predominantly formed a bcc structure, whereas increasing the Ni content to 25% resulted in a fully fcc structure.

In addition to the matrix composition, externally added second-phase particles, particularly Y_2_O_3_, can significantly alter the phase-transformation kinetics during MA [39]. To investigate the potential impact of Y_2_O_3_ on phase transformation behavior, Susila et al. [39], in their study on the fabrication of ODS austenitic steel (Fe-18Cr-8Ni-2W-0.25 Y_2_O_3_), found that the introduction of Y_2_O_3_ nanoparticles significantly accelerated the phase transformation process from the initial BCC ferrite to FCC austenite. The transformation rate was observed to be much higher than that of the Y_2_O_3_-free control sample. This phenomenon can be attributed to the dual role of Y_2_O_3_ particles during MA: On the one hand, acting as hard particles, Y_2_O_3_ effectively facilitated powder fracturing and grain refinement during ball milling, thereby enabling the rapid formation of a nanoscale grain structure. On the other hand, the grain refinement led to a significant increase in grain boundary density, while the incoherent interfaces between Y_2_O_3_ and the matrix further introduced a high density of lattice defects, thereby accelerating atomic diffusion. The aforementioned structural features collectively enhanced the solid-state diffusion and chemical homogenization of alloying elements such as Ni and Cr, ultimately significantly promoting the formation of the austenite phase. A study by Kumar et al. further corroborated this mechanism, indicating that the synergistic addition of Y_2_O_3_ and Ti could more effectively accelerate the formation process of the solid solution [40].

Whether a single fcc phase is formed is also contingent upon the milling intensity; specifically, higher milling energy tends to facilitate the emergence of the bcc phase [41,42]. This phenomenon can be interpreted by the fact that higher energy input introduces a greater density of lattice defects and higher strain energy, thereby providing a more potent driving force for the phase transformation. Post-milling annealing generally promotes the reversion of the mechanically induced bcc phase to fcc austenite, but the extent of reversion depends on the alloy composition and thermal history. Fujiwara and Ameyama [41] employed pre-alloyed SUS316L powder and mechanically milled it at 250 rpm using ball-to-powder ratios of 1.8:1 or 12:1 for approximately 200–400 h, followed by annealing at 550, 700, or 900 °C for 300 s. In contrast, Enayati et al. [36] used elemental Fe, Cr, and Ni powders with different Fe-Cr-Ni compositions, which were mechanically alloyed at a ball-to-powder ratio of approximately 6:1 with bowl and supporting-disk speeds of approximately 400 and 235 rpm, respectively, for approximately 50–60 h. The latter powders were subsequently annealed at 700 °C for 1 h and cooled either in air or in the furnace. Enayati et al. [36] reported that austenite formed during annealing of the Fe-18Cr-8Ni and Fe-15Cr-15Ni alloys, but partial transformation back to martensite occurred during subsequent cooling, resulting in a γ + α′ structure at room temperature. Thus, the two studies are not intrinsically contradictory; their different final phase constitutions can be attributed to differences in the initial powder state, alloy composition, milling parameters, annealing conditions, and cooling path. In summary, the phase transformation behavior of ODS austenitic steels during mechanical alloying constitutes a complex phenomenon governed by the interaction between chemical composition and physical processes. Research indicates that the matrix composition dictates the stability of the austenite phase, whereas the severe plastic deformation inherent to the mechanical alloying process acts as the trigger for the phase transformation.

#### 2.1.3. Strategies for Suppressing Powder Sticking During Ball Milling: Two-Step Milling and Process Control Agents

The application of mechanical alloying to austenitic steels presents an inherent challenge: the superior ductility of the austenitic matrix renders it highly susceptible to severe plastic deformation and cold welding under high-energy mechanical impact, thereby resulting in severe powder agglomeration or sticking to the milling media [43]. This phenomenon not only leads to a significant decrease in powder yield but also compromises the homogeneous mixing of constituents, consequently impairing the final material properties [44]. To systematically address this issue, researchers have developed a series of multi-level control strategies encompassing two primary aspects: process flow design and the addition of process control agents [45]. The incorporation of process control agents (PCA) during the MA process represents another pivotal technique for suppressing excessive cold welding of the powders [46].

To effectively mitigate the issue of powder sticking associated with the austenitic phase, an effective “two-stage ball milling” strategy was successfully developed by Gräning et al. [46] and has been widely applied. The core rationale of this method lies in decoupling the physical processes of dispersoid distribution and matrix austenitization. The typical processing route is as follows: the first stage involves blending ferritic base powders with Fe_3_Y and Cr elemental powders, followed by mechanical alloying in a ball mill to obtain ferritic ODS powders. Subsequently, in the second stage, elemental Ni powder and stearic acid (C_18_H_36_O_2_) are added to the milled powder for a short-duration milling to drive the solid-state phase transformation of the matrix from ferrite to austenite. Stearic acid, introduced as a carbon-containing PCA, effectively reduces the adhesion of Ni and the resulting austenitic powder particles to the milling container, thereby achieving a powder yield of 100% in the second stage. This approach has also been adopted in studies by Ganesh et al. [19] and Ninawe et al. [47]. This strategy utilizes the distinct processing characteristics of different matrix phases, thereby improving both the yield and powder homogeneity of the MA process.

PCAs are typically highly surface-active organic compounds. During ball milling, they adsorb onto the nascent and highly reactive fracture surfaces of the powders, forming a physical barrier layer that effectively balances the competition between particle fracturing and cold welding. Research by Shashanka and Chaira [32] has confirmed that stearic acid, acting as a typical carbon-rich PCA, can significantly refine powder particle size and inhibit agglomeration, thereby improving the homogeneity of the mechanical alloying process.

**Figure 3:**
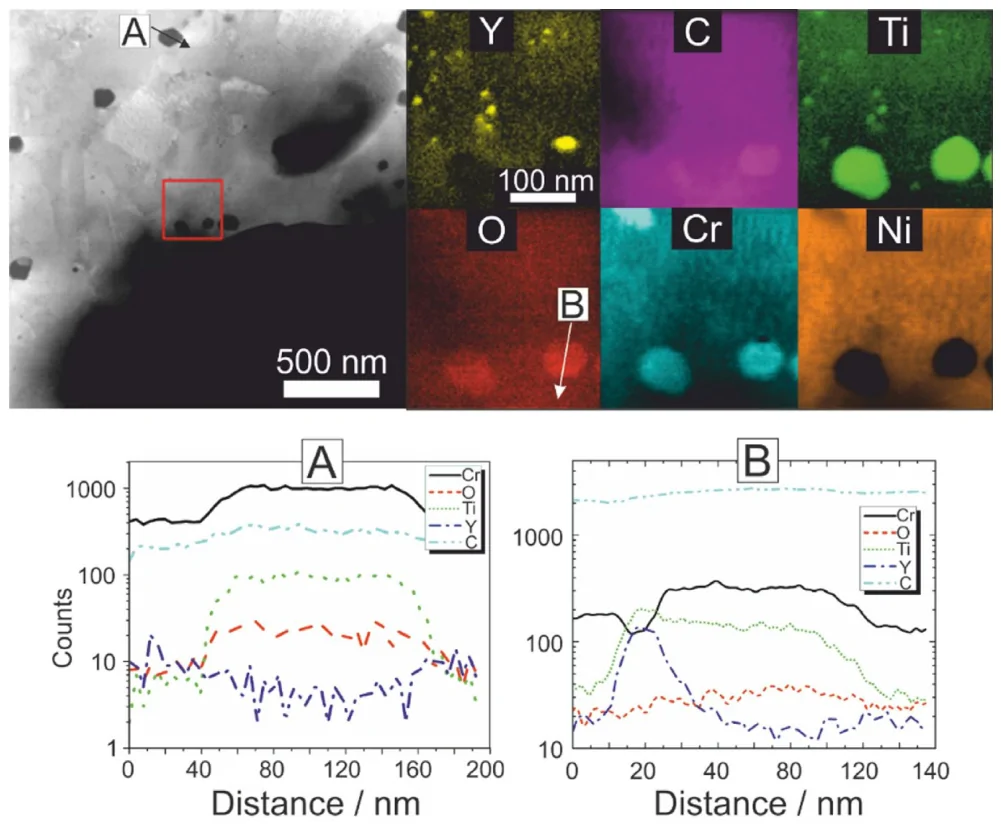
Effect of a carbon-containing process control agent on precipitate formation in an austenitic ODS steel after annealing at 1100 °C for 40 h. The HAADF-STEM image, EDX elemental maps, and line-scan analyses reveal coarse Cr-Ti-O-C-rich precipitates together with finer Y-Ti-rich particles. A and B indicate the positions corresponding to the EDX line-scan profiles. Reproduced without modification from Gräning et al. [46], under the applicable license.

However, the use of carbon-containing PCAs introduces a trade-off between improved powder processability and chemical contamination. Gräning et al. [46] investigated the precipitation behavior of an austenitic ODS steel produced by the two-step mechanical-alloying route described above. After the PCA-containing powder was annealed at 1100 °C for 40 h, coarse precipitates with diameters of approximately 100–200 nm were observed, as shown in Figure 3. EDX mapping and line-scan analyses revealed enrichment of Cr, Ti, O, and C in these coarse particles, indicating the formation of Cr(Ti)-O(C)-rich precipitates. In contrast, Y-rich precipitates smaller than approximately 20 nm showed a strong spatial correlation with Ti. These observations indicate that carbon introduced by stearic acid can alter the partitioning of Ti, allowing part of the Ti to participate in coarse carbon-containing precipitates rather than in the formation of fine Y-Ti-O nanoparticles.

Subsequent studies on the same PCA-assisted ODS austenitic steel further demonstrated that these precipitation characteristics evolve during consolidation and prolonged thermal exposure. Gräning et al. [45] observed Y–Ti–O nanoparticles together with coarse carbide-rich precipitates after hot extrusion and reported moderate oxide-particle coarsening during short-term annealing. In a later long-term study, Gräning et al. [25] found that the grain and oxide-particle structures remained comparatively stable during prolonged exposure at 700 and 900 °C, whereas annealing at 1100 °C resulted in pronounced oxide-particle coarsening, reduced particle number density, and extensive grain growth. These results indicate that the influence of carbon-containing PCAs extends beyond the milling stage and can affect the subsequent precipitation behavior and long-term microstructural stability of ODS austenitic steels.

**Figure 4:**
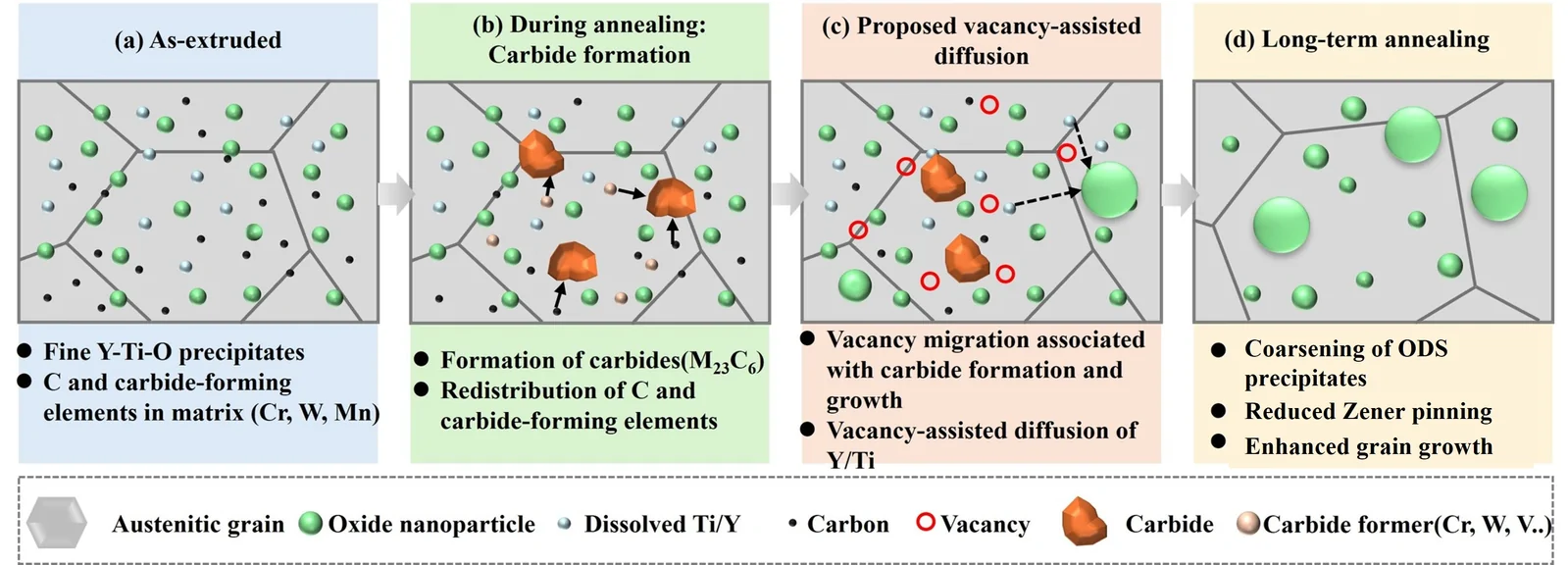
Schematic illustration of the proposed carbide-associated vacancy-assisted mechanism for oxide-particle coarsening, based on Ref. [25].

Kinetic analysis yielded an apparent activation energy of approximately 119–204 kJ mol^−1^ for oxide-particle coarsening between 900 and 1100 °C. This value is lower than the commonly reported activation energies for Fe self-diffusion in austenite and for the bulk diffusion of substitutional solutes such as Y and Ti [25]. On this basis, Gräning et al. [25] proposed that carbide formation and the associated vacancy flux may facilitate the migration of oxide-forming elements, thereby accelerating oxide-particle coarsening, as schematically illustrated in Figure 4. Nevertheless, this carbide-associated vacancy mechanism should be regarded as a proposed interpretation rather than a directly verified mechanism, because neither the vacancy evolution nor carbon-assisted diffusion of Y and Ti was measured directly.

More recently, Cao et al. [44] systematically investigated the effect of stearic-acid content on the mechanical alloying and subsequent consolidation of a Fe-15Cr-15Ni ODS austenitic steel. An addition of 0.2 wt.% stearic acid was insufficient to suppress cold welding and promote complete powder fracturing and elemental interdiffusion. Under their specific milling and hot-isostatic-pressing conditions, an addition of 0.6 wt.% provided a favorable balance among powder fracturing, cold welding, cleanliness, and elemental interdiffusion. The resulting steel contained predominantly fine Y_2_Ti_2_O_7_ nanoparticles, with an average size of approximately 5.67 nm, without pronounced coarse carbide or oxide inclusions. Further increasing the stearic-acid content to 1.0 or 1.5 wt.% increased the oxygen and carbon contents and promoted the formation of coarse oxides, carbides, oxycarbides, and possible prior-particle-boundary precipitates, thereby degrading microstructural homogeneity, mechanical properties, and subsequent processability. These results demonstrate that the effects of carbon-containing PCAs are strongly dependent on their concentration and the specific processing conditions. Therefore, the 0.6 wt.% value should be regarded as a system-specific optimum rather than a universally applicable PCA content.

Taken together, carbon-containing PCAs exhibit a process-dependent operating window. Insufficient additions may fail to suppress excessive cold welding, whereas excessive additions can increase contamination and promote coarse secondary phases. The PCA content should therefore be minimized while remaining sufficient for stable powder processing, and its optimum level should be determined according to alloy composition, milling conditions, and the subsequent consolidation route. Long-term microstructural stability should also be independently evaluated. In addition, to circumvent the detrimental effects associated with carbon contamination, Ganesh et al. [19] and Gräning et al. [43] proposed an innovative “contamination-free” alternative strategy: employing nitrogen (N_2_) gas as the PCA. This approach not only effectively mitigates the issue of powder sticking but also enables nitrogen, once dissolved in the matrix, to contribute to solid-solution strengthening and the stabilization of the austenite phase. Furthermore, during subsequent heat treatments, nitrogen can combine with elements such as vanadium (V) to form complex strengthening phases, thereby achieving a synergistic integration of process optimization and performance enhancement.

#### 2.1.4. Overview of Powder Consolidation Techniques

ODS austenitic steel powders prepared via MA exhibit metastable characteristics defined by high defect density and nanostructures; therefore, they must undergo subsequent consolidation to be transformed into dense bulk materials [48]. To achieve densification while simultaneously inhibiting grain growth and maintaining the stability of dispersoids, pressure-assisted high-temperature forming techniques have emerged as the primary processing routes [49]. Depending on the intended applications, sample dimensions, and fabrication efficiency, these consolidation techniques are primarily categorized into two classes. The first category encompasses HE and HIP, which are suitable for industrial-scale production; the second category features vacuum hot pressing (VHP) and SPS, which are tailored for rapid laboratory-scale fabrication [50].

For the fabrication of large-scale components and industrial-scale production, HE and HIP represent the most mature and widely applied technologies to date. HE represents a consolidation process that integrates the characteristics of hot pressing and thermomechanical processing. The simultaneous application of axial pressure and shear forces at elevated temperatures induces severe plastic deformation in the powders, thereby yielding fine-grained materials characterized by high density and low porosity. However, this technique entails significant limitations: the intense plastic flow causes grains to elongate along the extrusion direction, resulting in pronounced microstructural and mechanical anisotropy [51]. This phenomenon was corroborated by Suresh et al. [52], whose investigation revealed typical elongated grain morphologies in the longitudinal sections of the fabricated ODS austenitic steels. Furthermore, HE is generally associated with low efficiency and high cost; moreover, it often necessitates subsequent heat treatment to eliminate anisotropy and residual stresses.

In contrast, HIP employs high temperatures (950–1150 °C) and high isotropic gas pressures (100–200 MPa) to fabricate bulk materials characterized by exceptional density and microstructural homogeneity. Distinguished from HE, the HIP process precludes the formation of macroscopic texture, enabling the material to exhibit isotropy in both microstructure and mechanical properties, thereby significantly improving the synergy between strength and toughness [53]. By precisely regulating the sintering temperature (below the oxide coarsening threshold) and holding time (2–4 h), excellent metallurgical bonding between powder particles can be achieved [54]. Consequently, HIP has been widely adopted in studies by Zhou et al. [26,28,55,56,57,58,59], Leo et al. [6], and Peng et al. [60], establishing itself as a reliable choice for the fabrication of high-quality, high-performance industrial-grade ODS austenitic steels.

In laboratory-scale studies, VHP and SPS are frequently employed for the rapid evaluation of material properties, the exploration of new alloy compositions, and the consolidation of relatively small specimens because of their high densification efficiency and comparatively flexible processing conditions [61]. VHP promotes powder consolidation under uniaxial pressure in a vacuum environment, whereas SPS combines uniaxial pressure with pulsed electric-current heating. In particular, the rapid heating and short holding times of SPS can substantially reduce the overall consolidation duration [62] and are generally beneficial for limiting grain growth and retaining the fine microstructural features of mechanically alloyed powders compared with conventional HIP and HE routes [63]. Despite these advantages, the scale-up of SPS remains affected by several engineering constraints. The achievable component geometry and size are influenced by the graphite tooling, pressure-transfer configuration, and temperature distribution within the compact [64]. In addition, the requirements for high-current power supplies, vacuum or controlled-atmosphere systems, and cooling equipment increase the complexity and cost of the process. Carbon transfer from graphite dies or foils may also contaminate the consolidated material at elevated temperatures. Although diffusion barriers, such as tungsten foils or suitable coatings, can reduce direct carbon contact, they introduce additional processing complexity and may not completely eliminate contamination [65]. SPS/FAST has already been implemented in industrial production for several material systems; however, within the ODS austenitic-steel literature reviewed here, it has been used predominantly for the rapid consolidation of relatively small research specimens. Its broader application to ODS austenitic steels therefore requires improved control of tooling design, temperature uniformity, component geometry, processing cost, and carbon contamination [66].

VHP constitutes a consolidation process involving the simultaneous application of high temperature and uniaxial pressure within a vacuum environment. The applied external pressure accelerates inter-particle diffusion and pore closure, enabling difficult-to-sinter ODS alloys to attain a dense, fine-grained microstructure at relatively lower temperatures [39,67]. Susila et al. [39] successfully synthesized nanocrystalline ODS austenitic alloys exhibiting ultra-high strength via VHP, thereby validating the efficacy of this technique in preserving nanostructures. Although VHP possesses a lower heating rate compared to SPS, it offers a relatively uniform distribution of microstructural stress and is suitable for fabricating specimens of slightly larger dimensions. Its limitations, however, lie in the uniaxial direction of pressure application, geometrical constraints imposed by the die, and a persistent tendency for grain growth at elevated temperatures. To facilitate comparison among different fabrication routes, the representative processing parameters reported for ODS austenitic steels are summarized in Table 1. The studies are grouped according to the combination of powder preparation and consolidation techniques, including MA/RBM-SPS, MA-HIP, MA-HP/VHP, and MA-HE. The table separately lists the mechanical-alloying parameters and the subsequent consolidation conditions, thereby allowing the processing characteristics of different manufacturing routes to be compared more directly.

The parameter ranges in Table 1 reflect the different densification mechanisms of the consolidation routes. VHP and HP employ uniaxial pressure and were mainly used for exploratory Fe-Cr-Ni alloys at 900–1100 °C. HE relies on high-temperature plastic deformation, with extrusion temperatures generally close to 1070–1150 °C. SPS was applied to a broad range of alloy compositions at approximately 920–1050 °C and 50 MPa for only 5–15 min, whereas HIP was used predominantly for 304-, 316-, and 15Cr-15Ni-derived alloys containing approximately 0.35 wt.% Y_2_O_3_, typically at 1100–1150 °C and 100–200 MPa for 2–3 h. These ranges are representative and should not be regarded as universal processing windows because the optimum conditions depend on both alloy composition and equipment configuration.

### 2.2. Casting Methods

Although melting methods possess the distinct advantages of high scalability and cost-effectiveness, traditional metallurgical processes, such as casting, exhibit significant limitations in the fabrication of ODS steels. Due to the high thermal stability, low density, and poor wettability of oxide particles with the molten steel, these particles are prone to coarsening and agglomeration, particularly when nano-sized particles are introduced, which makes it difficult to achieve a uniform nanoscale dispersion [78]. Simultaneously, the flotation behavior of nanoparticles within the melt further exacerbates the inhomogeneous distribution, resulting in instabilities in both microstructure and properties. Consequently, researchers are dedicated to overcoming this bottleneck in liquid metallurgy. Efforts are focused on two primary dimensions: optimizing the introduction and dispersion strategies for ex situ particles [79], and developing in situ reaction synthesis technologies for oxides [9], thereby exploring novel casting pathways to realize the homogeneous dispersion of nano-phases.

#### 2.2.1. Introduction and Dispersion Strategies for Ex Situ Particles

The core technical challenge of the melting method lies in the high interfacial energy between nanoparticles and the metal melt, which precludes particle incorporation, as well as the susceptibility of nanoparticles to agglomeration and subsequent failure due to coarsening via Ostwald ripening in the high-temperature melt [8]. Addressing these bottlenecks in wettability and dispersibility, some studies have proposed the utilization of ultrasonic cavitation-based solidification. This technique leverages transient cavitation effects generated by high-intensity ultrasonic waves to break nanoparticle clusters and clean particle surfaces, thereby physically enhancing wettability and bonding [80,81,82]. In the field of electromagnetic levitation melting, Verhiest et al. [7,79]. aimed to develop a fabrication route for ODS steels based on electromagnetic levitation melting combined with rapid solidification processing (RSP); their technical evolution reveals the critical role of wettability control in casting processes. In early investigations [7], they attempted to directly add dry nano-Y_2_O_3_ powder into the high-temperature melt under crucible-free levitation conditions, attempting to utilize the strong electromagnetic stirring force to achieve particle dispersion. However, experiments demonstrated that mere physical stirring could not overcome the extremely high interfacial tension between the ceramic particles and the liquid steel, resulting in the formation of surface slag or micron-sized agglomerates rather than a nanoscale dispersion. To resolve this interfacial wetting issue, Verhiest et al. [79] subsequently proposed an optimized strategy of “colloidal pre-dispersion combined with active element modification.” Instead of direct dry powder injection, this method introduces yttria as a colloidal dispersion and adds metallic Y as a surfactant. Research confirmed that metallic Y preferentially segregates to the solid/liquid interface during melting, significantly reducing the interfacial energy and lowering the contact angle to below 90°, thereby achieving effective wetting and encapsulation of the oxide particles by the liquid steel.

This process, which combines chemical modification with colloidal pre-dispersion, successfully achieved homogeneously dispersed oxide particles at the laboratory scale, validating from a physico-chemical perspective that improving wettability is a prerequisite for realizing cast ODS steels. Although breakthroughs have been made regarding the mechanism of wettability improvement, the industrial application of this approach still poses severe challenges. The critical dependence of this technical route on rapid solidification rates renders it incompatible with the slow cooling regimes inherent to large-scale industrial ingots, inevitably leading to the coarsening and flotation failure of nanoparticles within the ingot center. Consequently, this method is currently confined to fundamental laboratory research or the fabrication of small-sized precision components, making it difficult to directly translate into mass production technologies for large structural components.

#### 2.2.2. In Situ Reaction Synthesis Technologies for Oxide Particles

Although the wettability of externally added oxide particles can be improved through colloidal pre-dispersion, active-element modification, or physical-field assistance, ex situ particle addition still faces inherent risks of re-agglomeration, flotation, and interfacial instability in molten steel [8]. These limitations have motivated the development of in situ reaction routes, in which oxide particles nucleate directly within the melt [83]. Because these particles form simultaneously with the metallic matrix, they can establish comparatively clean and thermodynamically stable interfaces, thereby helping to overcome the wettability limitations associated with the direct addition of pre-fabricated ceramic particles [84]. In recent years, several in situ fabrication strategies based on conventional casting processes have been developed through the control of reaction thermodynamics, mass transfer, and solidification conditions [85]. To address the challenges in traditional casting where active elements are difficult to fully oxidize and precipitates are prone to coarsening within the melt, researchers proposed a fabrication strategy based on oxygen carrier assisted in situ reaction. In the investigation of 9Cr-ODS steel, Hong et al. [83] pre-placed Fe_2_O_3_ powders acting as the active oxygen source at the bottom of the mold and utilized the turbulence generated during pouring to enhance mass transfer, which promoted the in situ reaction between the dissolved yttrium and the released active oxygen to generate oxide particles with an average size of approximately 1.1 μm. Building upon this, Zhuang et al. [9] applied this method to 15-15Ti austenitic ODS steel and observed that the particles obtained solely via the casting in situ reaction remained relatively large, with a size of about 10 μm, and were prone to agglomeration. To mitigate this issue, they further introduced cold rolling with an 80% reduction followed by recrystallization annealing, a process that mechanically fragmented and redistributed the coarse particles through severe plastic deformation. Ultimately, a refined dispersion with sizes ranging from 50 to 100 nm was achieved within the recrystallized matrix.

However, the oxygen carrier method exhibits distinct limitations, primarily its strict reliance on subsequent thermomechanical treatments involving large deformations to achieve the mechanical fragmentation and redistribution of the particles. This dependence on intensive post-processing not only significantly increases process complexity but also restricts the application potential of this method for thick-walled castings and complex-shaped components, which are incompatible with severe plastic deformation.

**Figure 5:**
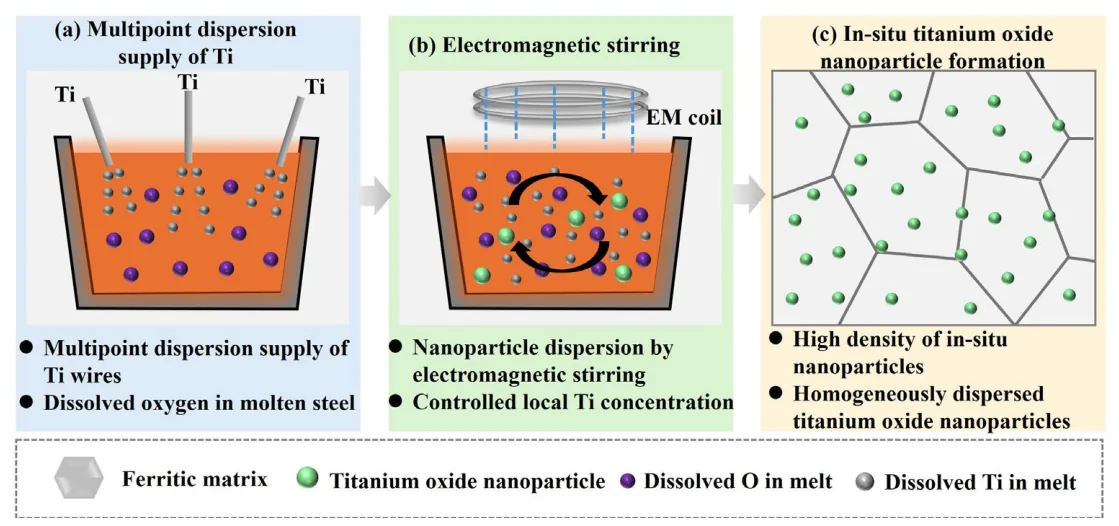
Schematic illustration of the in situ formation of titanium oxide nanoparticles through multipoint Ti supply and electromagnetic stirring, based on Ref. [84].

To surmount the limitations of the oxygen carrier method, which relies on severe post-processing for particle refinement, Tang et al. [84] proposed a more advanced internal oxidation strategy based on “multipoint dispersion supply combined with electromagnetic stirring,” as depicted in Figure 5. Distinct from methods relying on external oxygen sources, this approach utilizes the inherent dissolved oxygen within the melt and regulates nucleation kinetics by precisely controlling the addition mode of the active metal Ti. The core mechanism involves employing strong electromagnetic stirring with flow velocities reaching 10–20 m/s to rapidly disperse the Ti wires introduced via multipoint addition, thereby disrupting local high-concentration solute zones to significantly promote high-density nucleation while simultaneously inhibiting particle growth. Utilizing this method, Tang et al. successfully achieved the in situ formation of a large population of ultrafine titanium oxide particles with sizes smaller than 10 nm and as fine as approximately 8 nm within the ferritic steel matrix. These studies demonstrate that in situ reaction routes can produce finer particles and comparatively clean particle–matrix interfaces under controlled conditions. However, the available evidence remains limited, and several issues require further verification, including the sensitivity to alloy composition and processing parameters, the uniformity of particle distribution in large melt volumes, and the transferability of results obtained from ferritic steels to ODS austenitic steels. In situ reaction casting should therefore be regarded as a promising route for scale-up rather than an established preferred process.

Following the validation of this method’s feasibility, Tang et al. [86] further concentrated on the structural stability of the in situ generated nanoparticles under thermal environments, which serves as a critical factor determining their viability as effective substitutes for conventional strengthening phases. The investigation revealed that the in situ generated Ti_3_O_5_ nanoparticles exhibited excellent thermal stability. During tempering at 700 °C, their size coarsened only slightly from approximately 8 nm to 14 nm, while consistently maintaining a coherent or semi-coherent interfacial relationship with the matrix. Such stable interfacial characteristics effectively restrict atomic diffusion and interface migration, thereby averting the strengthening failure typically caused by the coarsening or dissolution of conventional precipitates at elevated temperatures. Consequently, the Ti_3_O_5_ nanoparticles can sustain significant strengthening effects in high-temperature environments, ensuring the thermal stability of the material properties. In summary, the casting technology based on in situ reaction, owing to its process controllability and superior strengthening and toughening effects, constitutes a preferred technical route for the future large-scale fabrication of high-performance ODS steels.

### 2.3. Additive Manufacturing

Additive manufacturing (AM) provides a direct route for producing geometrically complex ODS components and reduces the number of consolidation and machining steps required by conventional powder metallurgy [87,88]. Rapid solidification and controllable melt-pool conditions may also promote grain refinement and the formation of cellular substructures. However, AM does not inherently ensure a uniform oxide dispersion [89]. During laser processing, pre-added oxide particles may partially dissolve, coarsen, agglomerate, redistribute with melt-pool flow, or be lost through spattering [88,89]. The repeated thermal cycles can also introduce porosity, residual stress, columnar grain structures, and microstructural anisotropy [90]. The final oxide distribution and material properties therefore depend strongly on powder preparation, energy density, scanning strategy, atmosphere, remelting conditions, and subsequent heat treatment [91,92]. At present, research on additively manufactured ODS austenitic stainless steels mainly focuses on ODS 304 and ODS 316 systems, owing to their promising structural stability and high-temperature performance. Within this research framework, ODS 316L has been investigated from both theoretical and experimental perspectives. On the theoretical side, Wang et al. calculated the temperature-dependent diffusivities of Y, Ti, and O in molten 316L ODS steel using ab initio molecular dynamics [93]. They further coupled melt-pool thermal analysis, density functional theory, and cluster dynamics to predict the size distribution of Y–O nanoparticles under different LPBF parameters, and the predicted particle sizes showed good agreement with experimental measurements [94].

These studies provide important insight into oxide dissolution, solute redistribution, and nanoparticle precipitation during laser additive manufacturing. On the experimental processing side, the introduction and control of strengthening phases such as Y_2_O_3_ remain critical for fabricating ODS 304/316 stainless steels by laser-based additive manufacturing, particularly selective laser melting/laser powder bed fusion. Common powder modification strategies include mechanically coating nano- or submicron-sized Y_2_O_3_ particles onto the surface of 304L or 316L matrix powders through low-energy ball milling to promote oxide dispersion during laser melting [91,95], as well as introducing oxide precursors onto powder surfaces by in situ chemical coating or spraying methods to achieve localized selective doping [96]. During laser processing, the high-energy laser beam can partially dissolve pre-added oxides into the matrix and strongly affect melt-pool convection, thereby influencing solute redistribution, cellular substructure formation, and subsequent precipitation of nanoscale oxide particles. To suppress oxide agglomeration and promote nanoscale refinement and uniform dispersion, remelting strategies and laser parameter optimization, such as adjusting energy density and scanning speed, have also been explored. In addition, powder morphology optimization, including improved sphericity and surface coating quality, together with post-processing heat treatments, has been used to reduce strengthening-phase loss, particle agglomeration, and processing-induced defects. For example, heat treatment at an appropriate temperature, such as 1000 °C, can help retain the cellular substructures formed during laser processing while promoting fine particle precipitation, thereby improving the balance between strength and ductility. Overall, the available studies demonstrate the feasibility of fabricating ODS austenitic steels by AM, but the current evidence is based mainly on 304L- and 316L-type alloys and relatively small laboratory specimens. Differences in powder coating, oxide content, laser parameters, and post-processing also make direct comparison among studies difficult. AM should therefore be regarded as an emerging route with advantages in geometrical flexibility rather than as a process that has already surpassed conventional powder-metallurgy routes. Further work is required to quantify oxide loss during processing, improve build-to-build reproducibility, reduce defects and anisotropy, and evaluate high-temperature and creep performance in larger components.

### 2.4. Comparative Assessment of Fabrication Routes

The principal advantages, limitations, and current development status of the fabrication routes discussed above are compared in Table 2. This comparison is based on the studies reviewed in Section 2.1, Section 2.2 and Section 2.3 and is intended to identify the conditions under which each route is most applicable rather than to establish a universal ranking.

No fabrication route can be regarded as universally superior. Mechanical alloying followed by HIP or HE currently has the broadest experimental basis for ODS austenitic steels and generally provides comparatively reliable densification and oxide-dispersion control. The representative conditions summarized in Table 1 are approximately 1100–1150 °C, 100–200 MPa, and 2–3 h for HIP, and extrusion temperatures of approximately 1070–1150 °C for HE. SPS is commonly conducted at approximately 920–1050 °C and 50 MPa for 5–15 min, making it suitable for rapid alloy development, while VHP has mainly been applied to relatively small laboratory specimens. These ranges should not be interpreted as universally optimal processing windows because the appropriate conditions depend on alloy composition, oxide content, milling history, equipment configuration, and component geometry.

At present, HIP and HE may therefore be regarded as the leading research routes in terms of the amount of available experimental evidence rather than as universally optimal technologies. Conventional melting offers greater production capacity but remains limited by poor control of externally added oxide particles. In situ reaction casting and additive manufacturing provide potential advantages in scalability and geometrical flexibility, respectively, but their reproducibility, particle uniformity, and long-term performance require further verification.

## 3. Microstructural Characteristics

### 3.1. Grain Structure

**Figure 6:**
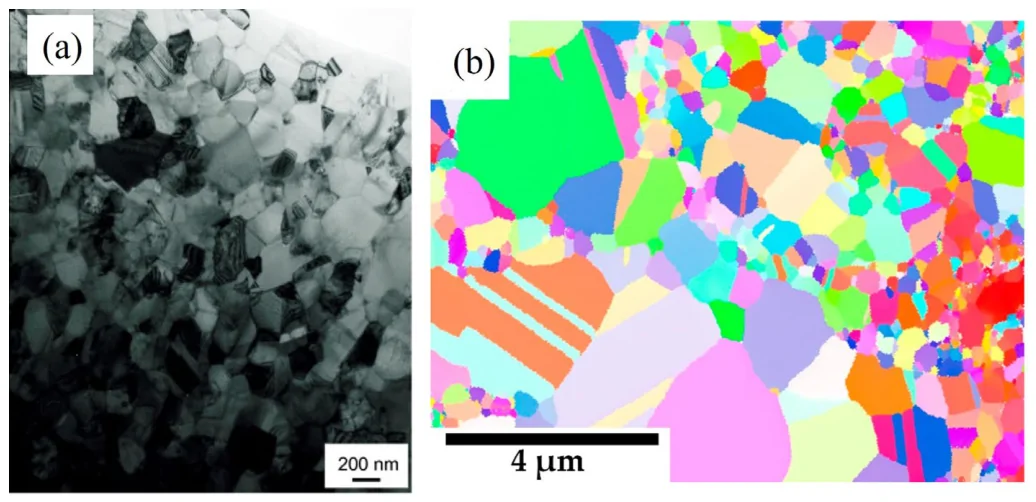
Representative grain structures of powder-metallurgy ODS austenitic steels: (**a**) TEM micrograph showing relatively equiaxed grains with an average size of approximately 300 nm in HIP-consolidated ODS austenitic steel [26]; and (**b**) EBSD IPF map showing a bimodal grain structure in SPS-consolidated ODS 316L steel [98].

The ultrafine grained (UFG) structure constitutes a distinguishing feature of ODS austenitic steels compared to their conventional counterparts. Attributed to the effective pinning of grain boundaries by dispersed oxide particles, grain growth is significantly inhibited during high-temperature consolidation or heat treatment [97]. Nevertheless, the final grain structure is governed by the combined effects of the mechanically alloyed powder state, oxide dispersion, and thermal history rather than by the consolidation technique alone. For example, Zhou et al. [26] fabricated a Fe-18Cr-8Ni-1Mo-0.5Ti-0.35Y_2_O_3_ ODS austenitic steel by mechanical alloying followed by HIP. The consolidated material exhibited relatively equiaxed grains with a mean size of approximately 300 nm, together with Y-Ti-Si-O-rich oxide particles smaller than approximately 20 nm, as shown in Figure 6a. The spatial distribution of oxide dispersoids is closely related to the homogeneity of the grain structure. Wang et al. [73] observed a pronounced bimodal grain structure in HIP-consolidated ODS-304 steel, in which fine Y-Ti-O-rich particles were concentrated in the small-grain regions, whereas almost no dispersoids were detected inside the large grains. These observations indicate that local variations in dispersoid density can produce substantial differences in grain-boundary pinning during consolidation. More recent observations in SPS-consolidated ODS 316L steels are consistent with this interpretation. As shown in Figure 6b, Pokorný et al. [98] reported a bimodal structure consisting of ultrafine grains up to approximately 0.6 μm and coarse grains up to approximately 2.8 μm. Regions enriched in Y_2_O_3_ exhibited distinctly finer grains because the oxide particles restricted grain-boundary migration.

**Figure 7:**
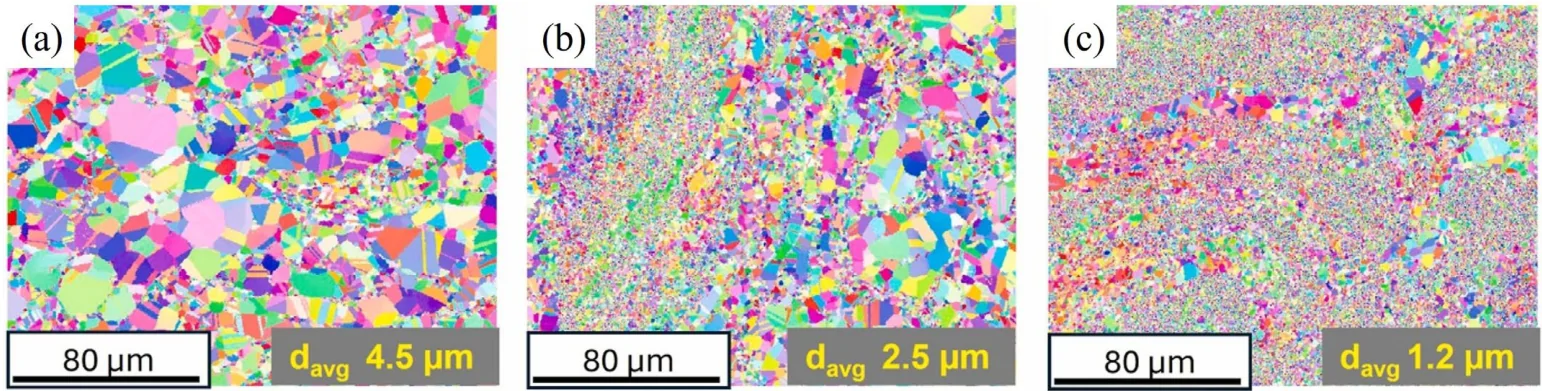
Evolution of the grain structure of 316L–1 wt.% nano-Y_2_O_3_ ODS steel with increasing mechanical-alloying time: EBSD IPF maps after mechanical alloying for (**a**) 5 h, (**b**) 10 h, and (**c**) 50 h, followed by pulse plasma sintering [99]. The different colors in the EBSD IPF maps indicate different crystallographic orientations; an IPF color key was not provided in the original source.

The mechanical-alloying history provides another important means of regulating the final grain structure. Dobkowska et al. [99] systematically investigated 316L ODS steels containing 1 wt.% nano-sized Y_2_O_3_ and subsequently consolidated by pulse plasma sintering. The corresponding EBSD IPF maps are shown in Figure 7. Increasing the milling time from 5 to 10 and 50 h reduced the average grain size from approximately 4.5 to 2.5 and 1.2 μm, respectively, while increasing the fraction of fine grains. The authors attributed this evolution to the combined effects of accumulated deformation, discontinuous recrystallization, and changes in the distribution of Y_2_O_3_ during mechanical alloying. These results demonstrate that the grain structure of powder-metallurgy ODS austenitic steels is established progressively during powder preparation and consolidation rather than solely during the final sintering step.

Although powder-metallurgy consolidation generally favors relatively equiaxed grain structures, subsequent directional thermomechanical processing can substantially modify the grain morphology. Severe plastic deformation may introduce processing-induced microstructural anisotropy in ODS steels [100], while directional solidification can likewise produce elongated or columnar grains [101]. In hot-extruded ODS austenitic steel, Suresh et al. [52] observed equiaxed grains with an average size of approximately 280 nm in the transverse section, whereas the longitudinal section exhibited grains elongated along the extrusion direction with a characteristic size of approximately 440 nm. These observations demonstrate that directional processing can alter both grain morphology and orientation, thereby contributing to anisotropic mechanical behavior.

**Figure 8:**
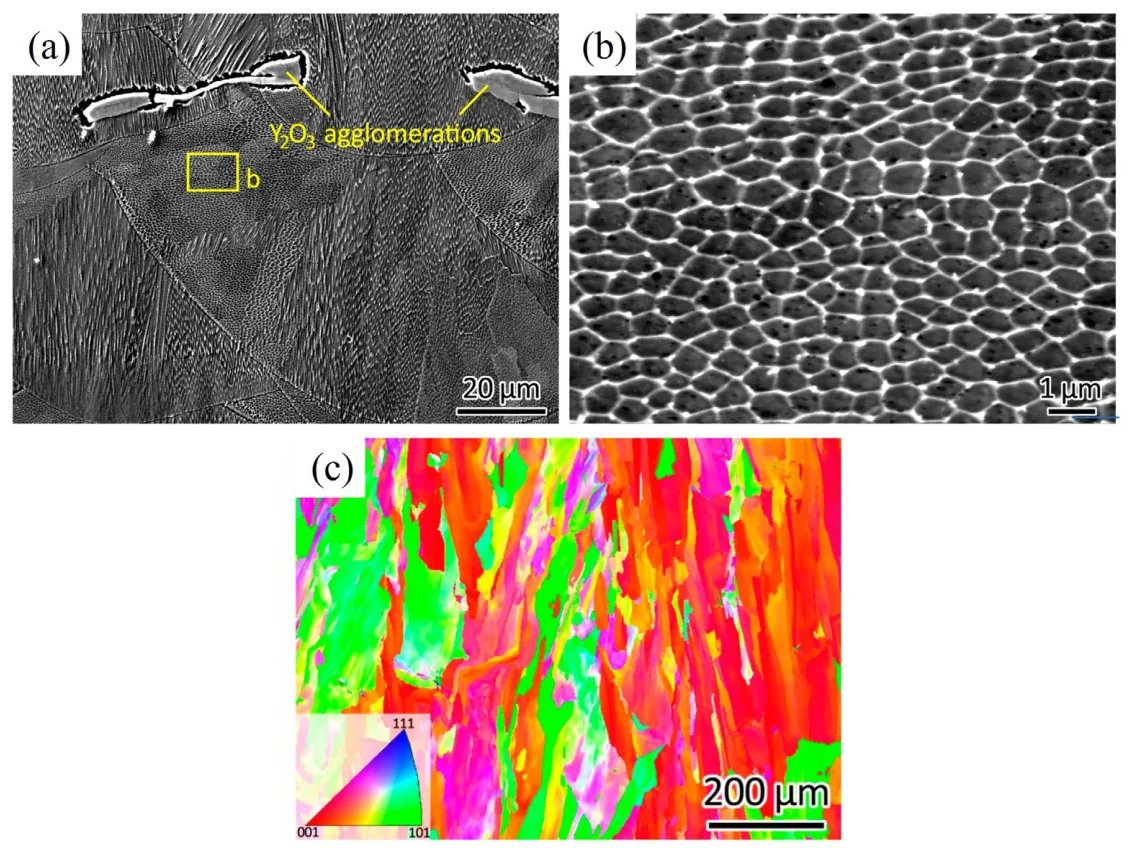
Hierarchical microstructure of LPBF-fabricated 316L–1.0 wt.% Y_2_O_3_ ODS steel: (**a**) overview of the cellular microstructure; (**b**) higher-magnification SEM image showing the cellular substructures; and (**c**) EBSD orientation map showing much larger stripe-like epitaxial grains [102].

Additively manufactured ODS austenitic steels exhibit a microstructural hierarchy distinctly different from that of powder-metallurgy materials. Zhai et al. [102] investigated LPBF-fabricated 316L-Y_2_O_3_ ODS steels and observed cellular substructures resulting from the high cooling rate inherent to LPBF. As shown in Figure 8a, cellular structures are distributed throughout the 316L-1.0Y_2_O_3_ matrix, while the higher-magnification image in Figure 8b reveals their submicrometer dimensions. The average cellular-structure sizes were approximately 0.49, 0.45, and 0.57 μm for 316L, 316L-0.3Y_2_O_3_, and 316L-1.0Y_2_O_3_, respectively. Importantly, these cellular structures should not be interpreted as crystallographic grains. EBSD analysis revealed much larger stripe-like grains produced by epitaxial growth, with a preferential <001> orientation along the build direction. As shown in Figure 8c, the 316L-1.0Y_2_O_3_ steel retained such directionally elongated grains, with an average crystallographic grain size of approximately 21.6 μm.

Notably, the introduction of Y_2_O_3_ did not produce pronounced crystallographic grain refinement in this LPBF system. The average grain sizes of 316L, 316L–0.3 Y_2_O_3_, and 316L–1.0 Y_2_O_3_ were approximately 25.9, 27.7, and 21.6 μm, respectively. Zhai et al. [102] attributed the limited grain-refinement effect primarily to the poor wettability between Y_2_O_3_ and molten 316L, which promoted nanoparticle agglomeration during LPBF. This behavior contrasts with the effective grain-boundary pinning commonly observed in powder-metallurgy ODS steels and indicates that the influence of oxide particles on grain structure depends strongly on their dispersion state and the thermal history associated with the fabrication route.

### 3.2. Dispersed Oxide Particles

Oxide dispersoids serve as the critical microstructural constituents underpinning the high-temperature strength and irradiation resistance of ODS austenitic steels [20]. Their chemical composition, crystal structure, size distribution, and interfacial characteristics with the matrix collectively govern the strengthening efficiency and thermal stability of the material [97]. Although Y_2_O_3_ serves as the predominant initial additive, following the intricate processes of mechanical alloying and high-temperature consolidation, the dispersoids ultimately stabilized within the matrix are typically not single-phase yttria, but rather complex oxides evolved through the synergistic interaction of multiple elements [58]. First-principles calculations using fcc γ-Fe as the matrix model indicated that Fe vacancies promote the association of Y and O atoms and may assist the early formation and growth of Y-O clusters [103]. This finding provides an atomistic basis for the possible role of the vacancy-rich mechanically alloyed austenitic matrix in oxide nucleation. In Ti-bearing systems, Y-Ti-O complex oxides constitute the primary strengthening phases. Among them, Y_2_Ti_2_O_7_ with a pyrochlore structure is widely identified as the dominant phase due to its superior thermodynamic stability [19,21,97]. While historical views distinguished Y_2_Ti_2_O_7_ as cubic and Y_2_Ti_2_O_5_ as orthorhombic, subsequent research has revealed that the partial substitution of Ti by Y facilitates a structural transition between these two phases [104]. In Si-containing systems, Y_2_O_3_ tends to react with Si in the matrix to form Y_2_Ti_2_O_7_. When reactive elements such as Al, Hf, or Zr are introduced, more intricate complex oxide systems evolve. For instance, in Al-bearing ODS austenitic steels, various Y-Al-O complex oxides can form, including monoclinic YAM (Y_4_Al_2_O_9_), garnet YAG (Y_5_Al_3_O_12_), hexagonal YAH (YAlO_3_), and perovskite YAP (YAlO_3_) [68,69]. Furthermore, the introduction of Hf or Zr promotes the formation of nanoparticles exhibiting higher structural complexity and superior high-temperature stability, such as fluorite-type Y_2_O_3_-HfO_2_ solid solutions, pyrochlore-structured Y_2_(Ti, Hf)_2_O_7_, and Y_4_Zr_3_O_12_ [23,58,105]. Beyond complex oxides, unique dispersoid architectures have been reported. For example, Peng et al. [60] discovered Cu-induced core–shell particles in which a Cu-rich phase acting as a heterogeneous nucleation core is encapsulated by Y_2_O_3_, while Mori et al. [72] observed the coexistence of Y_2_O_3_ and MnO nanoparticles in Ni-free, nitrogen-containing ODS steels. These diverse precipitation outcomes demonstrate that the precise regulation of minor alloying elements like Ti, Al, and Zr allows for the effective tailoring of dispersoid types and structures, thereby offering extensive compositional design space for optimizing the high-temperature thermal stability of the material.

**Figure 9:**
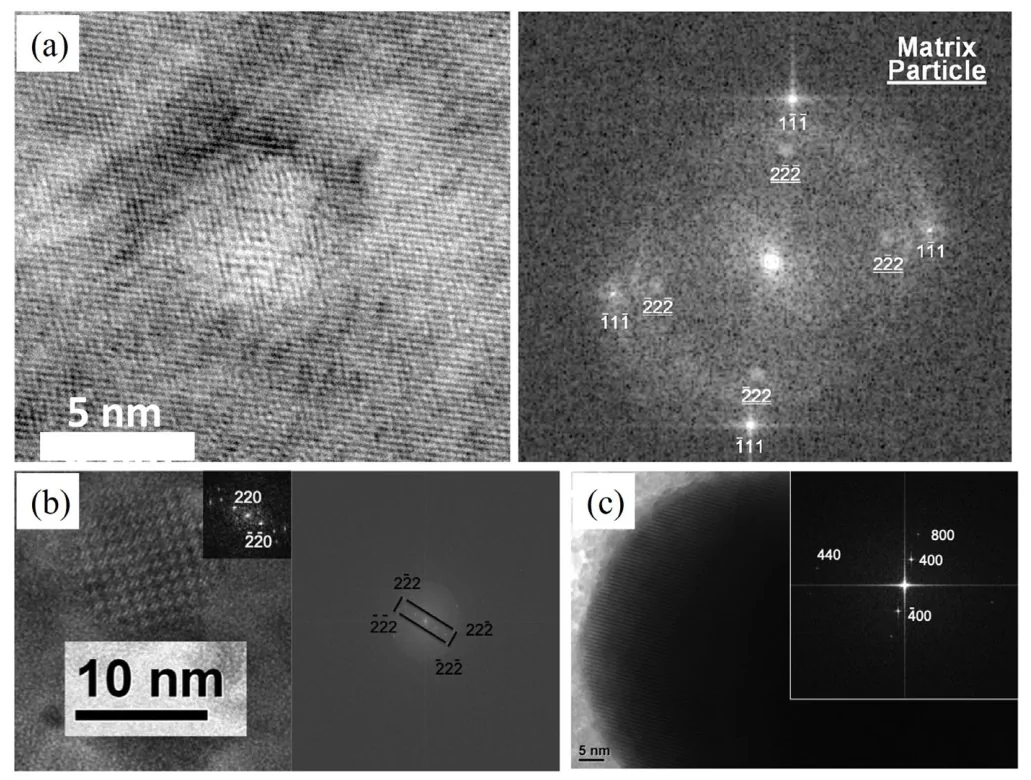
Representative oxide–matrix interfacial structures with different particle sizes in ODS austenitic steels: (**a**) HRTEM image and corresponding FFT pattern showing a nanoscale Y_2_Ti_2−x_O_7−x_ nanoparticle with a cubic-on-cubic (CoC) orientation relationship with the austenitic matrix [106]; (**b**) A semi-coherent interface in a medium-sized particle (~15 nm), where periodic misfit dislocations are introduced to accommodate the lattice mismatch; (**c**) An incoherent interface observed in a coarse particle (~40 nm), demonstrating a loss of specific orientation relationship due to significant growth [6].

The interfacial relationship between the dispersoids and the matrix, including coherent, semi-coherent, and incoherent configurations, is an important factor governing the strengthening behavior of ODS austenitic steels. Representative observations from independent studies indicate a general size-dependent evolution of the oxide–matrix interface, as summarized in Figure 9. Miao et al. [106] revealed that nanoscale Y_2_Ti_2-x_O_7-x_ particles in austenitic ODS steels could maintain a cubic-on-cubic (CoC) orientation relationship with the austenitic matrix. The HRTEM image and corresponding FFT analysis demonstrated a high degree of crystallographic matching between the oxide particle and matrix, as shown in Figure 9a. Miao [5] reported that particles with a size of approximately 15 nm exhibited semi-coherent interfaces containing periodic misfit dislocations that accommodated the accumulated lattice mismatch (Figure 9b). Leo et al. [6] found that coarse particles of approximately 40 nm formed incoherent interfaces and exhibited no distinct orientation relationship with the matrix (Figure 9c). Taken together, these observations suggest that particle coarsening is generally accompanied by a transition from coherent to semi-coherent and ultimately incoherent interfaces.

Interfacial coherency is not governed by particle size alone but is also influenced by the crystal structure, lattice mismatch, and orientation relationship of the oxide phase. Mao et al. [21] identified a semi-coherent relationship between Y_2_Ti_2_O_7_ and the austenitic matrix, with an orientation relationship of (220)Y_2_Ti_2_O_7_//(200)Matrix. Litvinov et al. [107] further reported that the approximately 6% lattice mismatch between face-centered-cubic Y_2_Ti_2_O_7_ and the matrix promotes the loss of full coherency during particle growth. In comparison, body-centered-cubic Y_2_O_3_ can exhibit a smaller lattice mismatch of approximately −2% along specific orientations and may therefore maintain a higher degree of coherency than Y_2_Ti_2_O_7_ particles of a comparable size.

The elastic strain field induced by interfacial mismatch plays a pivotal role in the mechanisms of material strengthening and irradiation resistance. Oka et al. [23] identified the presence of significant elastic strain fields surrounding the dispersed Y_2_Hf_2_O_7_ particles due to a large lattice mismatch. This strain field serves a dual function: on the one hand, it exerts a strong pinning effect on dislocation motion, thereby significantly contributing to dispersion strengthening; on the other hand, it acts as an effective sink for vacancies and interstitial atoms to regulate the migration and aggregation behavior of point defects, thus endowing the material with superior irradiation resistance [23]. Because of their high ductility, austenitic steel powders readily undergo cold welding and adhere to the milling media during mechanical alloying, promoting oxide-particle agglomeration and the formation of coarse dispersoids after consolidation, which can degrade the mechanical properties.

The microstructural differences produced by the various fabrication routes arise mainly from their distinct thermal histories, deformation modes, and mechanisms of oxide formation and transport. During powder metallurgy, mechanical alloying introduces a high density of defects and disperses the oxide-forming elements, while subsequent consolidation determines the grain morphology and residual defect structure. HIP applies nearly isotropic pressure and therefore generally favors comparatively equiaxed grains, whereas the directional plastic flow during HE produces elongated grains and texture. The rapid thermal cycle of SPS can restrict grain growth, although local temperature gradients and non-uniform oxide distributions may promote bimodal structures.

Melting-based routes are governed instead by particle wettability, buoyancy, melt convection, and solidification. Externally added oxides therefore tend to agglomerate, float, or segregate, whereas in situ reactions can promote particle nucleation within the melt and may produce cleaner interfaces. In additive manufacturing, steep thermal gradients and repeated melt-pool circulation promote cellular or columnar structures and can simultaneously dissolve, redistribute, or reprecipitate oxide particles. Despite differences among individual studies, the reported results consistently indicate that a fine and spatially uniform oxide dispersion stabilizes the grain structure, whereas oxide-depleted regions, coarse particles, and directional processing promote grain coarsening or anisotropy. Apparent differences among studies therefore generally reflect variations in oxide distribution, alloy composition, and thermal–mechanical history rather than intrinsically contradictory mechanisms.

## 4. Mechanical Properties

### 4.1. Synergistic Enhancement of Room Temperature Strength and Toughness

The traditional PM route, which combines MA with high-temperature consolidation, enables the fabrication of ODS austenitic steels featuring UFG microstructures, thereby imparting ultra-high strength to the material [97]. For instance, nanocrystalline ODS austenitic steels synthesized by Susila et al. [39] using MA coupled with VHP exhibited a macroscopic hardness as high as approximately 900 HV. It was indicated that grain boundary strengthening and dislocation strengthening constituted the dominant mechanisms, while dispersion strengthening also contributed a significant strength increment of approximately 347 MPa. Koncz et al. [71] fabricated ODS 316 steel via wet ball milling combined with SPS, achieving a three-point bending strength as high as 1363.6 MPa. Regarding tensile properties, Zhou et al. [26] produced ODS 304 steel using pre-alloyed powders consolidated by HIP, which exhibited an ultimate tensile strength of 1000 MPa at room temperature. However, this ultra-high strength state is frequently accompanied by a significant compromise in ductility.

The design of traditional high-strength materials is often constrained by the inverse correlation between strength and toughness, known as the strength-ductility trade-off, which constitutes a critical obstacle to the engineering application of advanced structural materials. Consequently, breaking through this performance boundary to achieve the synergistic enhancement of strength and toughness has emerged as a core objective in the field of ODS austenitic steels. Wang et al. [73] observed that the as-HIPed ODS-304 steel, despite possessing a high strength of 940 MPa, exhibited poor plasticity. This finding highlights the necessity of employing subsequent thermomechanical treatments to tailor the strength-ductility balance. The study demonstrated that following forging and hot rolling, the elongation was successfully increased to approximately 45% while maintaining a high strength of 920 MPa. Similarly, Zhao et al. [59] verified that heavy cold rolling combined with annealing at 1000 °C could increase the elongation of ODS 15Cr-15Ni steel by more than two-fold compared to the as-forged state. Collectively, these works indicate that for the traditional PM route, subsequent thermomechanical processing serves as a critical and effective pathway for regulating and optimizing the strength-toughness synergy of ODS steels.

**Figure 10:**
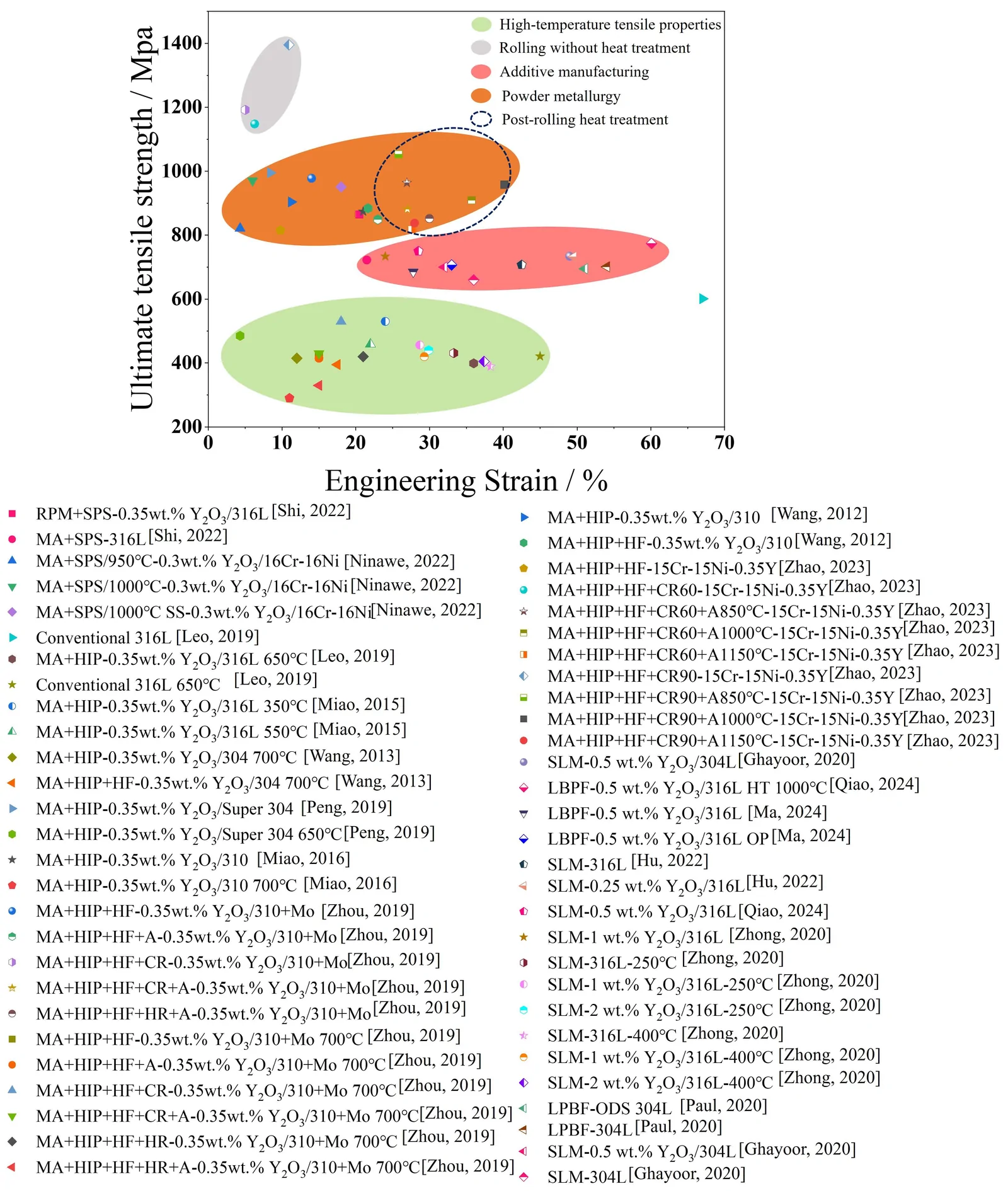
Summary of tensile properties of ODS austenitic steels fabricated via different processing routes. The data sources are indicated beside the individual alloy and processing conditions in the legend [5,6,9,26,35,47,55,57,59,60,73,74,76,91,95,96,108,109,110,111].

Advanced processes such as in situ synthesis and additive manufacturing have provided revolutionary pathways for fundamentally resolving the strength-ductility trade-off by constructing unique microstructures. As a related example demonstrating the potential of in situ oxidation, Tang et al. [84] employed a multipoint dispersion-supply and electromagnetic-stirring process to generate coherent or semi-coherent nano-Ti_3_O_5_ particles directly during the solidification of a ferritic steel, as described in Section 2.2.1. The resulting steel exhibited a yield strength of 711 MPa, an ultimate tensile strength of 810 MPa, and an elongation of 22%. Although this study was conducted on a ferritic steel rather than an ODS austenitic steel, it demonstrates the potential of controlled in situ oxidation for improving the strength–ductility balance and provides a useful processing reference for future ODS austenitic steel development. Additive manufacturing (AM), particularly selective laser melting (SLM/LPBF), has also made milestone progress in strengthening and toughening. Zhong et al. [95] fabricated ODS 316L steel via SLM, achieving an unprecedentedly high elongation of 91% alongside a high yield strength of 574 MPa. This work revealed for the first time a unique “delayed necking” toughening mechanism: during deformation, dispersed nanoparticles induce the homogeneous nucleation of numerous nano-voids throughout the sample, but the robust cellular substructure network characteristic of the SLM process effectively inhibits the growth and coalescence of these voids, thereby allowing plastic deformation to be uniformly distributed across the entire gauge length prior to fracture. Similarly, the SLM-fabricated 304L ODS austenitic steel reported by Ghayoor et al. [108] exhibited an ultimate tensile strength of approximately 700 MPa and an elongation of approximately 32%. The relatively high ductility was attributed to the work-hardening capacity associated with its micron-sized austenitic grains, with an average size of approximately 8.1 μm. Furthermore, Hu et al. [96] discovered that the introduction of Y_2_O_3_ during SLM promoted the formation of nano-twins, which, in synergistic interaction with dispersed precipitates and refined cellular structures, also successfully realized the simultaneous enhancement of strength and ductility in the 316L alloy. Figure 10 systematically summarizes the tensile property data of ODS austenitic steels fabricated via different processing routes, as derived from the available literature. Overall, the material strength exhibits an anticipated declining trend with increasing temperature. In terms of processing-route comparison, PM remains the predominant fabrication route and generally provides a more favorable overall combination of mechanical properties than AM, while subsequent thermomechanical treatment can further optimize the strength-ductility balance. Appropriate mechanical processing coupled with heat treatment significantly enhances ductility and toughness without compromising strength, thereby achieving a synergistic improvement in both properties. Conversely, if the material is subjected solely to rolling deformation without subsequent annealing, the ODS steel suffers from a severe deterioration in ductility despite exhibiting high strength induced by work hardening.

### 4.2. High-Temperature Strength and Creep Resistance

The core value of ODS steels resides in their superior high-temperature performance. Research indicates that the effective pinning of the microstructure by nanoparticles is crucial for maintaining high-temperature strength [107]. To gain a deeper insight into the strengthening mechanisms, researchers have utilized advanced techniques such as in situ synchrotron radiation XRD to investigate the load partitioning behavior of ODS steels during deformation. Miao et al. employed in situ synchrotron X-ray diffraction to investigate load partitioning between the austenitic matrix and different secondary phases in ODS 304, ODS 316, and ODS 310 steels [5,74,76]. These studies demonstrated that the load-bearing response of the dispersed phases depends on both particle type and particle size. In the ODS 310 steel investigated in Ref. [76], fine Y_2_Ti_2_O_7_ nanoparticles with an average diameter of approximately 4.6 nm coexisted with TiN precipitates that were generally larger than 100 nm. At room temperature, both phases carried higher stresses than the austenitic matrix, with Y_2_Ti_2_O_7_ exhibiting a slightly stronger load-partitioning response. As the testing temperature increased, the extra lattice strain of TiN decreased markedly and became lower than that of the matrix above 500 °C, indicating substantial degradation of its load-partitioning effect. In contrast, the fine Y_2_Ti_2_O_7_ nanoparticles retained a pronounced extra lattice strain at temperatures up to 800 °C. These observations highlight the comparatively persistent contribution of ultrafine oxide nanoparticles to the high-temperature strengthening of ODS austenitic steels. Regarding the contributions of distinct strengthening mechanisms, quantitative analysis has confirmed the synergistic action of multiple mechanisms. Research by Morrall et al. [30] on Zr-containing ODS 304L steel demonstrated that its high yield strength of 767 MPa primarily stemmed from grain refinement governed by the Hall-Petch effect, which contributed approximately 540 MPa; in contrast, precipitation strengthening derived from the Orowan mechanism contributed a relatively smaller amount of about 44 MPa. These quantitative studies provide a significant theoretical basis for property prediction and the integrated design of composition, processing, and properties for ODS steels. Seils et al. developed a model-based description of the temperature-dependent strength of austenitic and ferritic ODS steels from room temperature to 800 °C [24]. For the austenitic ODS steels, the analysis quantified the Hall–Petch, Orowan, and dislocation-strengthening contributions and associated the pronounced strength decrease above approximately 500 °C with the onset of creep-based deformation. Ghayoor et al. [87] systematically evaluated the high-temperature performance of L-PBF-fabricated ODS 304L steel and reported a yield strength of 290 MPa at 600 °C, more than twice that of conventionally annealed 304L steel (113 MPa). This enhanced high-temperature strength was attributed to the thermally stable cellular substructure pinned by in situ-formed Y-Si-O nanoparticles. In another study, Zhang et al. [112] employed computationally assisted alloy design to fabricate a dual-phase ODS steel that maintained high compressive strengths of 964 MPa and 672 MPa at 650 °C and 700 °C, respectively. These studies collectively point to a core strategy: stabilizing the unique cellular substructure inherent to additive manufacturing via nanophase pinning is key to achieving superior high-temperature strength. Kotan [29] reported that, for mechanically alloyed nanocrystalline Fe-18Cr-8Ni steel under the investigated annealing conditions, the addition of 1 at.% Zr or 1 wt.% Y_2_O_3_ limited grain coarsening during annealing at 1100 °C, retaining grain sizes below approximately 250 nm and a Vickers microhardness of approximately 510 HV0.025. These results demonstrate the beneficial effects of Zr or Y_2_O_3_ additions on the thermal stability of the specific alloy investigated. These results indicate that Zr or Y_2_O_3_ additions can improve the thermal stability of the investigated alloy under the specific processing conditions, whereas the applicability of this conclusion to alloys with different compositions or processing histories requires further verification. Because the available studies involve different alloy compositions, fabrication routes, loading modes, and testing temperatures, the reported elevated-temperature strength values are not directly comparable. Nevertheless, these studies consistently indicate that the retention of fine oxide particles and stable grain or subgrain structures is beneficial to elevated-temperature strength, although the magnitude of the contribution depends on the specific material and testing conditions. In addition to short-term strength, creep resistance is essential for evaluating the long-term reliability of ODS austenitic steels.

**Figure 11:**
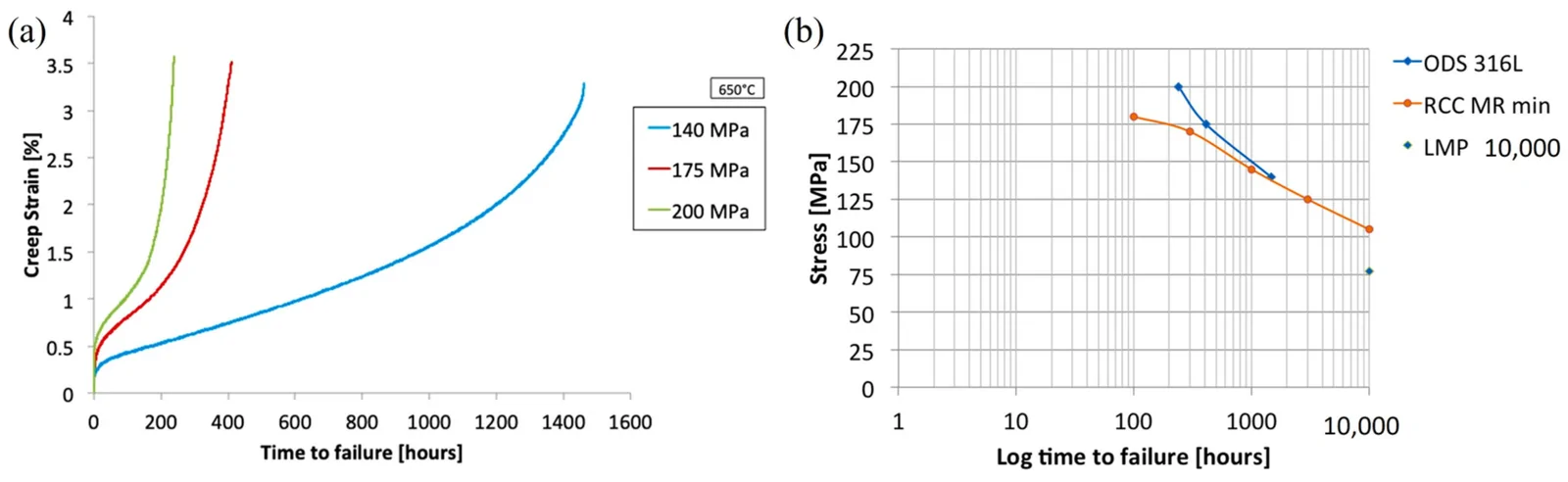
Creep behavior of ODS 316L steel at 650 °C. (**a**) Creep rupture test curves at different stress levels. (**b**) Plot of stress vs. log of time to failure; isolated point is the extrapolation for 10,000 h based on Larson–Miller parameter [6].

Compared with tensile-property data, creep data for ODS austenitic steels remain limited. Direct comparison among different studies is generally difficult because of differences in alloy composition, fabrication route, specimen condition, testing temperature, applied stress, and data-analysis method. The following discussion therefore focuses primarily on the internally consistent creep results reported for HIP-consolidated ODS 316L steel. Leo et al. [6] investigated the creep behavior of an ODS 316L steel fabricated by mechanical alloying and hot isostatic pressing at 650 °C under applied stresses of 140–200 MPa. As shown in Figure 11a, the material exhibited only a brief steady-state creep stage, followed by an extended tertiary creep stage that accounted for more than 50% of the total creep life. More notably, as shown by the Larson-Miller parameter (LMP) analysis in Figure 11b, although the data points for ODS 316L remained above the RCC-MR standard curve within the experimental testing range of less than 2000 h, indicating a certain degree of compliance, its stress-rupture life curve exhibited a steeper decline compared to the standard material. Extrapolation based on the LMP predicted a 10,000 h rupture strength of approximately 77 MPa, which is significantly lower than the minimum stress value required by the code, revealing a deficiency in long-term service capability. Microstructural analysis provided a possible explanation for the coexistence of relatively high short-term strength and limited long-term creep resistance: while the material retained some fine coherent or semi-coherent particles smaller than 15 nm that could effectively pin dislocations, it also contained a large number of coarse oxides exceeding 50 nm in size. HRTEM confirmed that these large particles held an incoherent relationship with the matrix and thus could not provide the “attractive interaction” based on the Rösler-Arzt mechanism, leading to a substantial reduction in the critical stress required for dislocation detachment. Simultaneously, the tendency of coarse particles to cluster at grain boundaries induced premature intergranular fracture, thereby significantly impairing the creep ductility and rupture life of the material. For the investigated HIP-consolidated ODS 316L steel, the results indicate that the beneficial effects of fine coherent or semi-coherent particles were partly offset by coarse incoherent oxides and particle clusters at grain boundaries, which were associated with reduced creep ductility and rupture life. These observations highlight the importance of controlling the particle-size distribution, oxide–matrix interface structure, and grain-boundary particle accumulation; however, the applicability of the reported particle-size ranges to other ODS austenitic steels remains to be established.

The available mechanical property data show that fabrication route alone does not determine the performance of an ODS austenitic steel. The tensile and creep responses are controlled by the combined effects of grain size and morphology, oxide size and number density, interface structure, dislocation or cellular substructure, secondary phases, porosity, and processing-induced anisotropy. Powder-metallurgy materials currently provide the broadest tensile-property database, and subsequent forging, rolling, or annealing can improve the strength-ductility balance by modifying the grain structure and work-hardening capacity. However, HE may introduce directional mechanical behavior, and excessive consolidation or heat-treatment temperatures may reduce strengthening through grain and particle coarsening.

SPS- and VHP-consolidated materials frequently exhibit high strength because of their fine grain structures, but the available ductility and long-term creep data remain limited. Additively manufactured 304L- and 316L-based ODS steels have shown promising room-temperature strength–ductility combinations associated with cellular substructures, nanotwins, and high work-hardening capacity. Nevertheless, these results are based on a limited number of alloy systems and cannot yet establish a general advantage over powder-metallurgy materials. Direct mechanical-property evidence for melting-based ODS austenitic steels is still insufficient. Consequently, reliable performance ranking requires comparisons under similar alloy compositions, specimen conditions, and testing parameters, and the present evidence does not support a universal ranking of fabrication routes.

## 5. Conclusions and Outlook

This review examined the main fabrication routes, microstructures, and mechanical properties of ODS austenitic steels. Powder metallurgy, melting-based processing, in situ oxide formation, and additive manufacturing were compared. Particular attention was given to mechanical alloying, process control agents, oxide–matrix interfaces, tensile properties, and creep behavior. The main conclusions and research needs are summarized below:

• Powder metallurgy currently provides the best control of oxide size and distribution. However, its complex processing, high cost, and limited component size restrict wider application. Melting-based and additive-manufacturing routes offer better scalability or shape flexibility, but oxide agglomeration, flotation, segregation, and uneven distribution remain major problems;
• Mechanical alloying of austenitic powders requires control of both cold welding and contamination. Two-stage milling and process control agents can improve powder yield and mixing. Carbon-containing agents should be used at the minimum effective level because excessive additions may promote carbide formation and reduce microstructural stability;
• Oxide strengthening depends on particle size, composition, distribution, and interface structure. Fine coherent or semi-coherent particles generally provide more effective strengthening than coarse incoherent particles. Ti, Zr, and Hf additions can modify oxide structure and stability, but their effects depend on alloy composition and processing conditions;
• ODS austenitic steels show good tensile strength at room and elevated temperatures, but creep data remain limited. More work is needed to clarify the effects of particle coarsening, grain-boundary damage, interfacial separation, and microstructural anisotropy during long-term service;
• Future studies should focus on process reproducibility and scale-up. Processing parameters, powder characteristics, oxide size and number density, grain structure, and testing conditions should be reported consistently. Long-term creep, fatigue, irradiation, and corrosion behavior should also be evaluated under relevant service conditions.

## Figures and Tables

**Table 1:** Processing parameters for the fabrication of ODS austenitic steels via powder metallurgy.

Composition (wt.%)	Processing	BPR	Rpm	Time (h)	Sintering Parameters			
					Pressure (MPa)	Temp (°C)	Time	Refs.
Fe-16.6Cr-10Ni-2.5Mo-0.5Ti-2.5Y_2_O_3_ Fe-16.6Cr-10Ni-2.5Mo-0.5Ti-2.5ZrO_2_	MA+HP	10:1	300	40	20	1000	1 h	[27]
Fe-14Cr-20Ni-2.5Mo-2.5Al-2Mn-0.5Y_2_O_3_ Fe-14Cr-20Ni-2.5Mo-2.5Al-2Mn-5Y_2_O_3_	MA+HP	30:1	500	16	50	1100	30 min	[68,69]
Fe-18Cr-8Ni-2W-0.25 Y_2_O_3_	MA+VHP	10:1	300	25	200	900	N.A.	[39]
Fe-16.8Cr-22Ni-2W-2.4Mo-1.5Mn-0.62Si-0.15La-0.2Ti	MA+HE	10:1	700	4	N.A.	1070	N.A.	[52]
PNC316+0.35 Y_2_O_3_+0.1Ti+0.6Hf	MA+HE	5.5:1	440	48	N.A.	1150	2 h	[70]
Fe-18Cr-11Ni-2.2Mo-0.9Si-0.35 Y_2_O_3_	RBM+SPS	6:1	300	36	50	1050	10 min	[35]
Fe-17Cr-12Ni-2.5Mo-2.0Si-1 Y_2_O_3_	MA+SPS	5:1	600	5	50	920	5 min	[71]
Fe-16 Cr-16 Ni-1.5W-0.21Ti-0.3 Y_2_O_3_	MA+SPS	10:1	-	32	50	1000	5 min	[47]
Fe-13Cr-20Mn-0.5N-0.35 Y_2_O_3_	MA+SPS	5:1	300	40	50	950 1000	5 min 15 min	[72]
Fe-18Cr-8Ni-1Mo-0.5Ti-0.35 Y_2_O_3_	MA+HIP	10:1	380	60	120	1100	3 h	[26]
Fe-18Cr-8Ni-1Mo-0.5Ti-0.35 Y_2_O_3_	MA+HIP	5:1	300	30	100	1100	2 h	[73]
304/316/310+3 Y_2_O_3_+3Ti	MA+HIP	5:1	300	50	100	1100	2 h	[11,55]
Fe-15Ni-15Cr-0.35Y and Fe-15Ni-15Cr-0.35 Y_2_O_3_	MA+HIP	10:1	300	30	120	1150	2 h	[58]
Fe-18Cr-8Ni-2W-1Ti-0.35 Y_2_O_3_	MA+HIP	10:1	380	60	200	1150	3 h	[28]
Fe-16.82Cr-13.23Ni-2.48Mo-0.2N-0.3Ti-0.35 Y_2_O_3_	MA+HIP	5:1	300	30	100	1150	3 h	[5]
Fe-18Cr-8Ni-1Mo-0.5Ti-0.15Si-0.35 Y_2_O_3_	MA+HIP	5:1	300	30	100	1100	2 h	[74]
Fe-17Cr-13Ni-2.5Mo-0.7Si-0.4Mn-0.35 Y_2_O_3_-0.5Ti	MA+HIP	5:1	300	30	100	1150	3 h	[75]
Fe-24Cr-18Ni-1.9Mo-0.3Ti-Y_2_O_3_	MA+HIP	5:1	300	30	100	1150	3 h	[76]
316L-0.3Ti-0.35Y_2_O_3_	MA+HIP	5:1	300	30	100	1150	3 h	[6]
Fe-18Cr-8Ni-3Cu-0.4Nb- Y_2_O_3_	MA+HIP	6:1	200	N.A.	120	1150	3 h	[60]
Fe-13Cr-16Ni-2Al-0.35 Y_2_O_3_	MA+HIP	5:1	300	30	100	1100	N.A.	[77]
Fe-15Cr-15Ni-2.4Mo-0.5Zr-0.35Y	MA+HIP	10:1	300	30	120	1150	2 h	[59]

**Table 2:** Comparative assessment of fabrication routes for ODS austenitic steels.

Fabrication Route	Main Advantages	Main Limitations
MA followed by HIP or HE	Relatively controllable oxide dispersion; high densification; broad experimental basis	Complex and costly multistep processing; powder contamination; HE-induced microstructural anisotropy
MA followed by SPS or VHP	Rapid consolidation; effective retention of fine-grained structures; flexible conditions for alloy screening	Tooling and component-size constraints; temperature gradients; possible carbon contamination during SPS; geometrical limitations associated with uniaxial pressure
Conventional ex situ melting	Compatibility with established melting facilities; relatively low cost; potential for large-volume production	Poor wettability of externally added oxides; particle agglomeration, flotation, segregation, and coarsening
In situ reaction casting	Reduced dependence on the wettability of externally added particles; potential for fine particles, clean interfaces, and scale-up	Strong sensitivity to reaction and solidification conditions; uncertain particle uniformity in large melts; limited direct evidence for ODS austenitic steels
Additive manufacturing	Direct fabrication of complex geometries; rapid solidification; reduced machining requirements	Oxide dissolution, redistribution, agglomeration, or loss; porosity, residual stress, columnar grains, and microstructural anisotropy

## Data Availability

No new data were created or analyzed in this study. Data sharing is not applicable to this article.
